# Timing of nest vegetation measurement may obscure adaptive significance of nest‐site characteristics: A simulation study

**DOI:** 10.1002/ece3.2767

**Published:** 2017-01-25

**Authors:** Mark D. McConnell, Adrian P. Monroe, Loren Wes Burger, James A. Martin

**Affiliations:** ^1^ Department of Wildlife, Fisheries and AquacultureCollege of Forest ResourcesMississippi State UniversityMississippiMSUSA; ^2^ Forest and Wildlife Research CenterMississippi State UniversityMississippiMSUSA; ^3^ Warnell School of Forestry and Natural ResourcesSavannah River Ecology LabUniversity of GeorgiaAthensGAUSA; ^4^ Present address: Adrian P. Monroe, Natural Resource Ecology LaboratoryColorado State UniversityFort CollinsCOUSA

**Keywords:** grasslands, logistic exposure, measurement bias, nesting ecology, nest‐site selection, simulation, vegetation structure

## Abstract

Advances in understanding avian nesting ecology are hindered by a prevalent lack of agreement between nest‐site characteristics and fitness metrics such as nest success. We posit this is a result of inconsistent and improper timing of nest‐site vegetation measurements. Therefore, we evaluated how the timing of nest vegetation measurement influences the estimated effects of vegetation structure on nest survival. We simulated phenological changes in nest‐site vegetation growth over a typical nesting season and modeled how the timing of measuring that vegetation, relative to nest fate, creates bias in conclusions regarding its influence on nest survival. We modeled the bias associated with four methods of measuring nest‐site vegetation: Method 1—measuring at nest initiation, Method 2—measuring at nest termination regardless of fate, Method 3—measuring at nest termination for successful nests and at estimated completion for unsuccessful nests, and Method 4—measuring at nest termination regardless of fate while also accounting for initiation date. We quantified and compared bias for each method for varying simulated effects, ranked models for each method using AIC, and calculated the proportion of simulations in which each model (measurement method) was selected as the best model. Our results indicate that the risk of drawing an erroneous or spurious conclusion was present in all methods but greater with Method 2 which is the most common method reported in the literature. Methods 1 and 3 were similarly less biased. Method 4 provided no additional value as bias was similar to Method 2 for all scenarios. While Method 1 is seldom practical to collect in the field, Method 3 is logistically practical and minimizes inherent bias. Implementation of Method 3 will facilitate estimating the effect of nest‐site vegetation on survival, in the least biased way, and allow reliable conclusions to be drawn.

## 1 Introduction

Nest success has been identified as a crucial population parameter for birds (DeMaso et al., 2011; Hoekman, Mills, Howerter, Devries, & Ball, 2002; Wisdom & Mills, 1997). The prevailing paradigm suggests that birds select nest sites based on proximate cues such as vegetation structure that are linked to ultimate factors that confer fitness (e.g. nest success) (Block & Brennan, 1993; Hilden, 1965; Martin, 1993; Wiens, 1989). As such, much of the literature on nest success has focused on relationships between nest‐site characteristics and nest fate (Chalfoun & Schmidt, 2012). These studies assume that identification of habitat correlates of nest success will elucidate mechanisms such as predation and resource availability that shape adaptive resource selection. Furthermore, characterization of relationships among vegetation structure, patch characteristics, landscape context, and nest fate will inform our understanding of the evolutionary and ecological influences that shape life history strategies (Lack, 1968; Southwood, 1977) and subsequently that of species abundance and persistence (Martin, 1993; Pimm, Jones, & Diamond, 1988).

Nest predation is the most pervasive cause of nest failure in birds (Martin, 1995; Ricklefs, 1969), and the nest concealment hypothesis suggests that denser foliage reduces predator efficiency, thus increasing the probability of nest survival (Martin, 1992). Previous research indicates that nest sites providing greater visual obstruction or concealment (e.g. taller grass, denser grass, greater canopy cover) can lower predation risk of ground nesting species (Davis, 2005; DeLong, Crawford, & DeLong, 1995). However, copious literature exists that fails to establish congruence between habitat use and fitness. This ambiguity in research conclusions could be because of a multitude of factors associated with the complex process of predation. It also has been attributed to the wide variety of anthropogenic, methodological, and ecological‐evolutionary explanations (Chalfoun & Schmidt, 2012).

We posit a simple methodical explanation for the lack of detected congruence between nest vegetation and nest success. We hypothesize that habitat variables at nest sites are measured at improper times to properly capture ecological phenomena. Furthermore, inconsistent timing of habitat measurement among studies likely contributes to a non‐unified theory on nesting ecology. Ideally, we should measure nest‐site vegetation at the temporal scale that aligns with adaptive selection processes, if selection is indeed adaptive (Hilden, 1965). Given that nests are typically located during laying or incubation stages and vegetation structure may not be reliably measured without influencing nest fate (Götmark, 1992), many researchers delay vegetation measurement until nest fate (success or failure) is determined (Martin & Guepel, 1993; Lusk, Smith, Fuhlendorf, & Guthery, 2006; Dion, Hobson, & Lariviere, 2000; Pleasant, Dabbert, & Mitchell, 2006; Arredondo et al. 2007). Assuming vegetation changes in a predictable fashion, structurally and compositionally throughout the breeding season, conventional vegetation sampling protocols might contribute to bias in analyses and could lead to spurious conclusions regarding relationships between nest vegetation characteristics and nest fate.

To illustrate, consider two nests, with a 28‐day nesting period, initiated on the same day in structurally identical vegetation. If nest #1 is depredated on day 12, conventional protocol would be to measure nest‐site vegetation on this day. In contrast, if nestlings in nest #2 survive to completion, conventional protocol would be to measure nest‐site vegetation after nestlings have left the nest (day 28 or 29). During the 2 weeks between nest #1 failing and nest #2 completing, considerable changes in vegetation structure (height, visual obstruction, etc.) and/or composition might have occurred, especially in grassland systems. However, based on terminal vegetation measurements the researcher would have concluded that the nest that hatched occurred in taller, denser vegetation. Therefore, the risk of drawing an erroneous conclusion regarding the effects of surrounding vegetation on nest fate might exist (i.e. shorter vegetation of nest #1 reduced nest concealment and made it more vulnerable to predation than nest #2), when the effect of shorter vegetation is really an artifact of the timing of vegetation sampling. Had nest #2 also been sampled on day 12 (but still hatched on day 28), a similar measurement between nests may have been observed, perhaps discounting the previous conclusion. However, such an approach is often logistically impractical due to concerns for observer effects because the collection of nest vegetation measurements while a nest is still active could influence nest fate. A potentially less biased comparison would require an alternative protocol where sampling occurs at a consistent point in the nesting period for both nests, regardless of fate, for example sample nest #2 after completion and sample nest #1 on the estimated (expected) day of completion (i.e. day 28), therefore permitting a direct comparison between nests that were successful and unsuccessful. This approach could also elucidate structural or compositional differences in vegetation between nests that are not confounded by predictable vegetation growth over time.

The peer‐reviewed literature contains many examples of papers using the conventional protocol (Arrendondo, Hernandez, Bryant, Bingham, & Howard, 2007; Dion et al., 2000; Lusk et al., 2006; Martin & Guepel, 1993; and Pleasant et al., 2006) and is largely depauperate of the alternative protocol (notable exceptions include Sveum, Edge, & Crawford, 1998; Watters et al. 2002). The published research clearly illustrates the lack of an accepted timing for measuring nest‐site vegetation (Borgmann 2010, Borgmann & Conway, 2015). Borgmann and Conway (2015) conducted a review of 106 published studies regarding the nest concealment hypothesis. Thirty‐seven studies measured vegetation 1 week after fate (successful or failed), 51 after being inactive (vague description of timing in the published literature), and 19 not reported—indicating a wide discrepancy in reporting of methodology. They also found a relationship between the timing of foliage density estimates and support for the nest foliage density hypothesis; thus, methodology is obfuscating the potential underlying mechanisms driving adaptive selection and survival for birds. Borgmann and Conway (2015) also discussed possible solutions to the timing of vegetation measurements, but quantifiable solutions were beyond the scope of their work.

Our goal was to estimate the bias associated with conventional and novel methods for measuring nest‐site vegetation in an effort to build a consensus on when nest vegetation should be measured. The methods explored were as follows: Method 1—measuring at nest initiation; Method 2—measuring at nest termination regardless of fate; Method 3—measuring at nest completion for successful nests and at estimated completion for unsuccessful nests [a possible solution offered by Borgmann and Conway (2015)]; and Method 4—measuring at nest termination regardless of fate and incorporating nest initiation date as a covariate (ad hoc approach). We acknowledge that Method 1 is likely impractical for researchers to collect due to observer effects, but we included this method to compare nest measurements collected at a consistent time in the nesting cycle (Methods 1 and 3) and measurements with inconsistent timing (Methods 2 and 4). We used a simulation approach to estimate and compare bias among methods when modeling daily nest survival, basing our simulations on common effects of vegetation on nest survival and a common life history strategy.

## 2 Methods

### 2.1 Vegetation growth simulation

**Figure 1:**
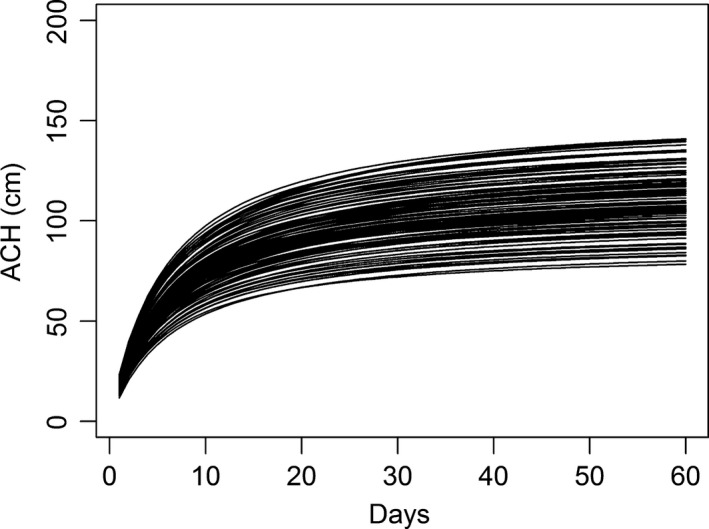
Simulated average canopy height (ACH) measurements across typical grassland bird nesting season

We simulated phenological changes in grass structure across a typical nesting season for grassland nesting birds (e.g. ~60 days). Simulation allowed us to illustrate scenarios while controlling for other confounding variables, whereas using real nests to collect vegetation measurements with the three methods (at initiation, at failure, and at completion) would likely compromise nest survival estimates through observer effects. Simulation also permits comparing known “true” effects to estimated effects from each method. We modeled average canopy height (ACH) for the study period using empirically derived growth data from native warm season grass fields monitored every two days and simulated this growth structure with multiple growth curves using the Michaelis–Menten equation (Figure 1). The Michaelis–Menten function is widely used in ecological investigations to model plant growth (Harper, O'Neill, Fielder, Newsome, & DeLong, 2009; Pacala, Canham, Silander, & Kobe, 1994). This equation is a monotonic function that asymptotically approaches saturation (Bayliss, 1985) and is appropriate for modeling vegetation growth which is rapid and linear early in the growing season when plants are allocating more energy to vertical growth, and then reaches an asymptote as growth slows and more energy is used for inflorescence production (Garnier, 1992). We used the Michaelis–Menten function as our deterministic equation to simulate ACH for each nest *i* as a function of time *t*:

$${\text{ACH}}_{\mathrm{it}}=\frac{at}{b+t}$$

where *a* is the asymptote height at which vegetation growth is maximized, *b* is the slope value when *a* is half its value, and *t* is the independent variable “*day*.” We simulated ACH_*it*_ using the Michaelis–Menten equation with a normal error structure on parameters *a* and *b* to generate a variety of growth curves to approximate stochasticity associated with vegetation growth. All simulations were performed in R (R Development Core Team 2014).

### 2.2 Nest survival simulation

Species respond differently to nest vegetation height based on differences in nesting ecology and life history strategies. Therefore, we modeled the bias associated with each method when modeling daily nest survival by fixing the coefficient of ACH_*it*_ (β_1_) at 3 levels, each with positive and negative effects, and at 0 (i.e. ±0.1, ±0.2, ±0.3, and no effect). These fixed coefficients represent the “true” effect of ACH on nest survival probability in our simulated datasets. We therefore calculated bias in our models as the absolute difference between the estimated effect of ACH and the simulated “true” fixed effect. For example, consider a model with a user‐defined “true” effect (+0.1) of ACH on daily nest survival probability. This value is on the logit scale and equates to a 10% increase in daily survival rate. If the logistic exposure model estimates an effect of 0.3, then the bias would be calculated as the absolute difference between the two values [i.e. |(0.1–0.3)| = 0.2)]. These simulated effects cover a range of relationships for species that show positive (e.g. northern bobwhite *Colinus virginianus*; Taylor, Church, & Rusch, 1999; Lusk et al., 2006; greater sage‐grouse *Centrocercus urophasianus*; DeLong et al., 1995) and negative effects of ACH_*it*_ (e.g. grasshopper sparrow *Ammodramus savannarum*; Patterson & Best, 1996; red‐winged blackbirds *Agelaius phoeniceus*; Caccamise, 1977; Adams, Burger, & Riffell, 2013). In addition, nests initiated earlier in the breeding season may experience greater success compared to nests initiated later (Perrins, 1970); however, species often exhibit a distribution of nest initiations over a breeding season with differential effects on nest fate. Therefore, we simulated nests with “Early” and “Late” nesting strategies by skewing the distribution of initiation dates across the 60‐day season. As such, “Early” nests were normally distributed around the first half of the season (with mean initiation 15 days before the middle of the season, or *skew* = –15) and “Late” nests were normally distributed around the last half of the season (*skew* = 15) (Appendix 1). Nesting studies for precocial birds (e.g. northern bobwhite, grouse (*Centrocercus spp*.), and turkeys (*Meleagris spp*.)) typically estimate survival for the incubation periods only, whereas survival is usually estimated for the combined laying and incubation periods for altricial birds. To minimize ambiguity, we modeled survival over the “nesting period” which could include incubation for precocial birds and incubation plus laying for altricial birds. We modeled a 28‐day nesting period to cover the range of incubation and laying periods for multiple species (Conover, Dinsmore, & Burger, 2011; Lituma, Morrison, & Whiteside, 2012; Williams, Austin, & Peoples, 1980). For each nest survival analysis, we simulated 600 nests over the study period with an intercept (β_0_) corresponding to a mean nest success rate 35% for a 28‐day nesting period, before incorporating effects of ACH.

Our simulation began by generating a random initiation date for each nest *i* as date_*i*_ ~ *N*(μ_date_, σ_date_), where μ_date_ = 30+ *skew* and σ_date_ = 30/7. This date was restricted to positive values and rounded to an integer. For each nest, we randomly simulated an ACH growth curve (ACH_*it*_; *see* Vegetation Growth Simulation), and the nest was randomly initiated at some time *t* along this growth curve. Once initiated, nest survival from *t* – 1 to *t* was modeled as a Bernoulli process with probability φ_*it*_, given that the nest was active at *t* – 1, that is, *y*_*it*_ ~ Bern(φ_*it*_ *|y*_*it–*1_ = 1). We included covariate effects on survival as:

$$\text{logit}\left(\varphi_{\mathrm{it}}\right)=\beta_{0}+\beta_{1}{\text{ACH}}_{\mathrm{it}}+\varepsilon_{i}$$

where additional error for each nest was modeled as ε_*i*_ *~ N*(0, 0.05). Nests were considered successful if they survived until fledging age (28 days old). To simulate the encounter history, for each nest we randomly assigned an age of entry as age_*i*_ ~ *N*(μ_age_, σ_age_), where mean age (μ_age_) was the middle of the nesting cycle (*L*/2) and σ_age_ = *L*/6. If a nest failed before their entry age, they were not represented in the final sample of simulated nests. Thus, although 600 nests were initially simulated, <600 nests were available in each scenario for modeling nest survival. We then generated an encounter history for each nest, assuming 3‐day intervals between each visit beginning on the day of entry. For each nest, we recorded ACH_*it*_ when the nest was initiated (Method 1), ACH_*it*_ when the nest became inactive (Methods 2 and 4), and ACH_*it*_ at fledging age (Method 3). As such, ACH_*it*_ for Methods 2 and 4 was always less than ACH_*it*_ for Method 3 for failed nests but equal for fledged nests. We created 14 scenarios, one for each β_1_ coefficient for ACH, and generated 100 random datasets for each scenario.

We used the logistic exposure method (Shaffer, 2004) to model daily nest survival probability for each simulated dataset and created five models, including one model for each method and a null (intercept‐only) model. We also used Akaike's information criterion (Burnham & Anderson, 2002) to rank models for each dataset and calculated the proportion of simulations in which each model (nest measurement method) was selected as the best model (i.e. lowest AIC score). This approach allowed us to determine how often the most biased model is considered the “best” model, thus illustrating the potential for drawing erroneous conclusions. All modeling and simulations were performed in R (R Development Core Team 2014), and example code for simulation, nest survival modeling, and summary output is provided in Appendix 1.

## 3 Results

**Figure 2:**
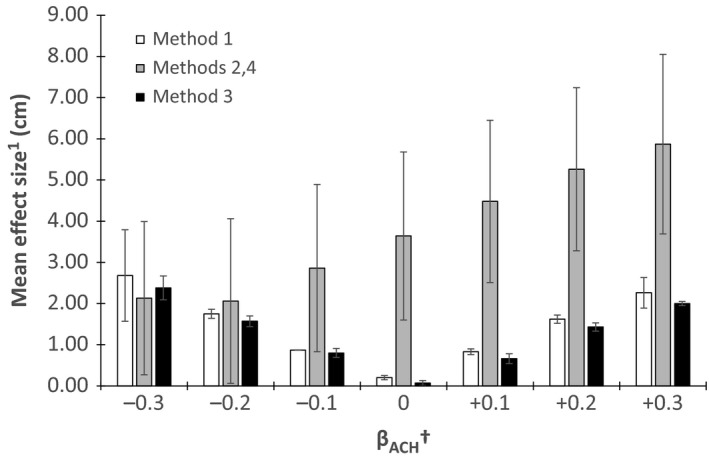
Effect size (as the absolute difference between ACH measurements for successful and unsuccessful nests) in average canopy height and standard error between successful and unsuccessful nests with varying coefficients for average canopy height for four nest vegetation measurement methods (Method 1 = measurement at nest initiation; Methods 2 and 4 = measurement at nest attempt completion (fledge or fail); Method 3 = measurement at estimated fledge date). ^†^User‐defined coefficient for effect of average canopy height on nest success

Of the 600 nests initially simulated in each dataset, the number of nests used in survival analysis varied (mean = 340.3; range: 310–371). ACH measurements for successful and failed nests varied by method, effect sign and size, and mean nest initiation date (Figure 2). Methods 1 and 3, where nest‐site vegetation was measured at a consistent timing regardless of nest fate (initiation for Method 1 and completion/estimated completion for Method 3), produced similar ACH measurements between hatched and failed nests. Methods 2 and 4, where vegetation was measured at inconsistent periods (hatch or failure), produced the greatest difference in ACH between hatched and failed nests because vegetation at failed nests was measured earlier than hatched nests. The absolute difference in ACH measurements between hatched and failed nests with Method 2 increased as the positive effect of ACH increased but not with greater negative effects of ACH (Figure 2).

### 3.1 Bias

**Figure 3:**
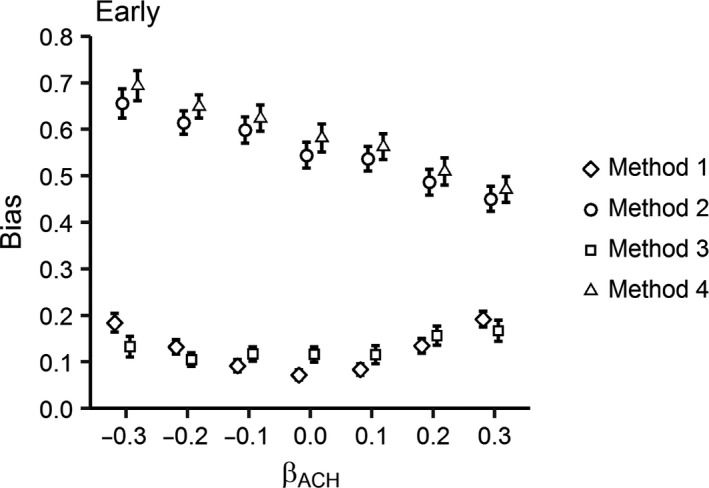
Bias (absolute difference between the simulated effect of ACH and the estimated coefficient from each model) in the effect of average canopy height (ACH) across a range of simulated coefficients [β = ±0.3, ±0.2, ±0.1, 0] for four nest vegetation measurement methods (Method 1 = measurement at nest initiation; Method 2 = measurement at nest attempt completion (fledge or fail); Method 3 = measurement at estimated fledge date; Method 4 = measurement at nest completion (fledge or fail) plus covariate for Initiation date) for Early‐initiated nests with 28‐day incubation periods and 35% nest success. Error bars represent 95% confidence intervals

**Figure 4:**
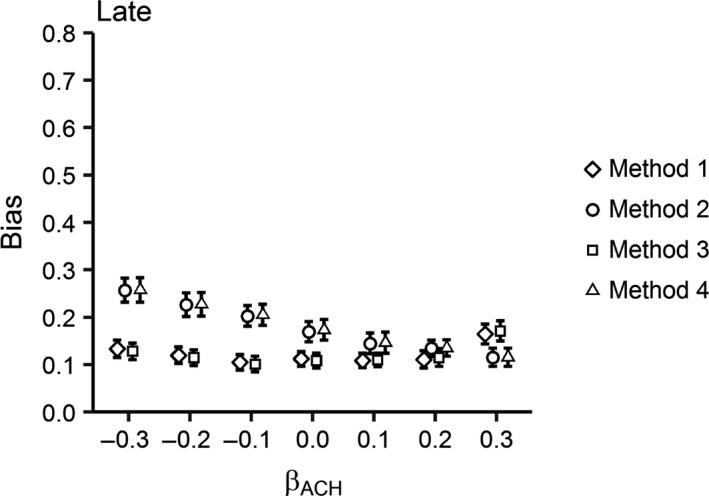
Bias (absolute difference between the simulated effect of ACH and the estimated coefficient from each model) in the effect of ACH across a range of simulated coefficients [β = ±0.3, ±0.2, ±0.1, 0] for four nest vegetation measurement methods (Method 1 = measurement at nest initiation; Method 2 = measurement at nest attempt completion (fledge or fail); Method 3 = measurement at estimated fledge date; Method 4 = measurement at nest completion (fledge or fail) plus covariate for Initiation date) for Late‐initiated nests with 28‐day incubation periods and 35% nest success. Error bars represent 95% confidence intervals

Methods 2 and 4 were the most biased, while Methods 1 and 3 were equally the least biased across all scenarios (Figures 3 and 4). The only exception was for a + 0.3 effect of ACH on daily nest survival probability, where Method 2 was the least biased for Late‐initiated nests. In this scenario, Method 3 was the most biased method (Figure 4). Across all methods, bias was less for Late‐initiated nests. Overall, bias was relatively constant for Methods 1 and 3 across all nesting scenarios. However, bias decreased with an increasing positive effect and increased with increasing negative effect of ACH on nest survival probability for Methods 2 and 4.

### 3.2 Model selection

**Figure 5:**
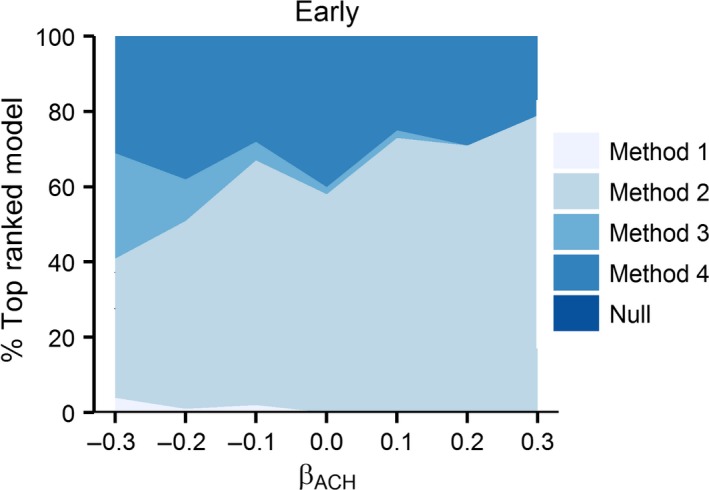
Proportion of simulations that four nest measurement methods (Method 1 = measurement at nest initiation; Method 2 = measurement at nest attempt completion (fledge or fail); Method 3 = measurement at estimated fledge date; Method 4 = measurement at nest completion (fledge or fail) plus covariate for Initiation date) and null model were chosen as the top ranked model, based on lowest AIC value across seven effects of average canopy height on nest survival ACH [β = ±0.3, ±0.2, ±0.1, 0] for Early‐initiated nests with 28‐day incubation periods and 35% nest success

**Figure 6:**
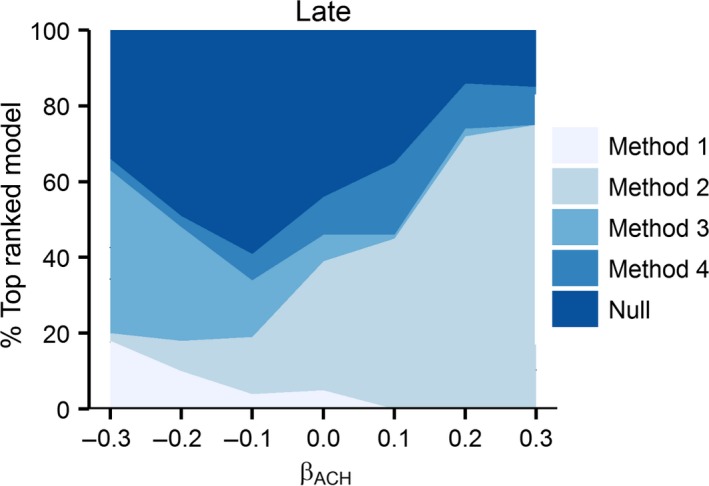
Proportion of simulations that four nest measurement methods (Method 1 = measurement at nest initiation; Method 2 = measurement at nest attempt completion (fledge or fail); Method 3 = measurement at estimated fledge date; Method 4 = measurement at nest completion (fledge or fail) plus covariate for Initiation date) and null model were chosen as the top ranked model, based on lowest AIC value across seven effects of average canopy height on nest survival ACH [β = ±0.3, ±0.2, ±0.1, 0] for Late‐initiated nests with 28‐day incubation periods and 35% nest success

Method 2 was overwhelmingly selected as the best approximating model with greater frequency across all scenarios with the exception of −0.3 effect of ACH on daily nest survival probability where Method 3 was selected in the majority of scenarios (Figures 5 and 6). For all Early‐initiated nests, the Method 4 was the second most often chosen model. The null model was generally the second most often chosen model for Late‐initiated nest when Method 2 was the most often chosen, and vice versa. One exception to this trend occurred for −0.2 effect of ACH on nest survival probability where Method 3 was the second most often chosen model after the null model. These results illustrate the preponderance of risk of drawing spurious or erroneous conclusions regarding nest‐site vegetation and nest fate.

The risk of drawing erroneous or spurious conclusion is present in all approaches to measuring nest‐site vegetation; however, overall Methods 2 and 4 were the most biased approach in 93% of modeling scenarios. Interestingly, Method 2 was also selected as the best model in 10 of those scenarios (71%). For scenarios with no simulated effect of ACH on nest fate, Method 2 estimated a bias >0.5 for Early‐initiated nests, indicating a strong positive or negative effect when the true effect was 0 (i.e. no effect). Furthermore, Method 2 was selected as the best competing model for this scenario. Therefore, not only was a spurious effect found with this method but model selection analysis indicated this method as the best model. This would lead to erroneous conclusions regarding the effect of vegetation structure on nest survival probability. Similarly, for the overwhelming majority of other effects of ACH, Method 2 or 4 was the most biased and most frequently chosen best model for all Early‐ and Late‐initiated nesting scenarios. Bias was less for all Late‐initiated scenarios, regardless of method. Unfortunately, the least biased Methods (1 and 3) for these scenarios were rarely selected as best models.

## 4 Discussion

Our objective was to evaluate and demonstrate how different timings of nest vegetation measurement methods influence conclusions regarding effects of nest vegetation structure on daily nest survival probability. Our simulation approach provides conclusive evidence of inherent bias associated with each of these vegetation measurement methods; however, the magnitude of bias varied with changes in the effect and sign of simulated ACH effect and nest initiation date. For most nesting scenarios simulated, the most biased method (Method 2) was chosen as the best competing model, whereas the least biased methods (1 and 3) were seldom selected as the best model. This result was unexpected considering the bias associated with Method 2. Further investigation revealed a correlation between ACH and nest exposure length when using Methods 2 and 4 that did not occur when using Methods 1 and 3. This correlation is representative of the fact that, on average, failed nests have shorter exposure periods and shorter vegetation, when using Method 2. When vegetation is measured at the time of failure instead of consistently across all nests, a positive relationship between vegetation height and exposure length is created. We hypothesize this correlation operates as a proxy for some other time‐varying process (i.e. survival time), therefore explaining a greater amount of variation and being selected as the top competing model. This inherent correlation provides further evidence that Method 2 can lead to a spurious conclusion based on correlative, rather than causal relationships.

The timing of vegetation measurement and its impacts on interpretation of selective processes is well documented (Burhans & Thompson, 1998; Rivera et al. 2009). Burhans and Thompson (1998) recommended measuring nest‐site vegetation when nest‐site selection occurs but acknowledge the risk of influencing nest fate with this approach. However, they found that measuring nest‐site vegetation later in the season did not affect the relationship between nest concealment and nest fate. Rivera et al. (2009) found that measurements of vegetation structure in highly seasonal ecosystems could be delayed until the end of the reproductive cycle. We found decreased bias in all Late‐initiated scenarios which is likely a result of the relatively flat slope of the vegetation growth curve during the Late nesting season where differences in ACH are minimal between hatched and failed nests. As grasses reallocate more energy to inflorescence production and less to growth, ACH becomes relatively stable; thus, the disparity in ACH between hatched and failed nests is reduced. Therefore, the inherent bias associated with timing of vegetation measurement could be minimized for Late season nesting attempts in grasslands but not in other environments (Rivera et al. 2009).

Whereas the validity of the nest concealment hypothesis is not in question, inherent methodological inconsistencies in field studies can hinder the ability to impartially investigate such and related hypotheses regarding avian habitat selection and nest survival (Borgmann & Conway, 2015). Multiple researchers have found a lack of congruence between factors that influence selection and those that affect nest success. For example, Davis (2005) found that grassland passerines selected nests with taller and denser vegetation, but these variables did not influence nest success. Similarly, Clark and Shutler (1999) found complete incongruence between nest‐site characteristics and nest fate for blue‐winged teal (*Anas discors*), gadwall (*Anas strepera*), and northern shoveler (*Anas clypeata*), but found some congruence for American wigeon (*Anas Americana*) and mallard (*Anas platyrynchos*). Considering the paucity of our understanding regarding the relationship between nest‐site selection and fitness and the multiple hypotheses for the discordance therein (Chalfoun & Schmidt, 2012), a more formal experimental investigation into methodological inconsistencies is warranted. Our results provide two primary contributions to nesting ecology research: (1) an additional, plausible explanation for the scarcity of consistent published effects of vegetation structure on nest fate (see Chalfoun & Schmidt, 2012), and (2) an alternative protocol for future nesting studies. We demonstrated the prevalence and magnitudes of bias associated with conventional protocols and illustrate a method that minimizes bias and thus provides more reliable estimates. Our results offer future studies a methodological standardization to evaluate alternative hypotheses without temporal bias in vegetation measurements. We also provide a method to reduce the ambiguity regarding timing of nest‐site vegetation measurements that is widespread in the literature (Borgmann & Conway, 2015).

While we provide some evidence of potential risks associated with measuring hazard process covariates at time of failure, more work is needed in this field. Such risks may become more apparent in rapidly changing environments or environments that change at a faster rate than can be measured. Modeling effects of changing vegetation on daily nest survival probability is another advance that should be explored, but currently remains unfeasible due to observer effects. An alternative would be to include a model for latent vegetation growth in conjunction with the nest survival model, informed by appropriate vegetation data, to more directly estimate the effects of vegetation structure on daily survival probability (N. T. Hobbs, personal communication). Converse, Royle, Alder, Urbanek, and Barzen (2013) provided a template for this approach by modeling a temporally varying, nest‐specific covariate (biting insect counts) on daily nest survival. They used incomplete data to spatially interpolate an index of daily insect abundance and its effect on nest success. Their approach provides a methodological model that could be adjusted for vegetation growth data at the nest site. Modeling temporally varying individual covariates with incomplete data could provide increased understanding of factors affecting both nest‐site selection and nest fate.

We did not model all possible effects of vegetation structure on daily nest survival nor did we attempt to model every functional relationship between vegetation structure and fate. We also only used a single life history to demonstrate the phenomena. We chose our model parameters from the available literature and use them only as a conceptual model to illustrate the inherent bias in conventional methodology. However, we acknowledge that potential bias could be variable among other life history strategies. For example, nesting period length could result in differential bias. Shorter nesting periods would reduce the disparity between vegetation measurements of hatched and failed nests therefore possibly reducing bias, and vice versa. Similarly, bias could be affected by the magnitude of nest success, strength of relationship (±) with vegetation metrics, and any interaction of these variables. We encourage researchers to build upon our base model to answer more specific questions regarding methods in nesting ecology. Finally, we acknowledge that our results may produce uncertainty regarding previous research findings. However, our intention is not to disregard any published estimates but rather to provide a quantifiable, reproducible, and realistic approach to aid researchers in choosing the best field methods for future research.

We can conclude the risk of estimating spurious relationships between nest vegetation and daily nest survival is greater when using Method 2, measuring at nest termination regardless of fate; Method 2 also is a common method reported in the literature. We can also conclude that the risk of drawing erroneous conclusions is prevalent for Early‐ and Late‐initiated nests, but the risk is greater for Early nests. Methods 1 and 3 are, on average, similarly less biased than Method 2. However, Method 1, measuring at nest initiation, is usually logistically impractical for many field studies where nesting individuals are susceptible to abandonment if disturbed near the onset of initiation. Therefore, implementing Method 3 where nest‐site vegetation is measured at a consistent point in the nesting period regardless of nest fate will facilitate modeling effects of nest vegetation on daily survival probability in the least biased way and allow reliable conclusions to be drawn.

This research was funded by the Forest and Wildlife Research Center and Department of Wildlife, Fisheries, and Aquaculture at Mississippi State University and the Warnell School of Forestry and Natural Resources. We thank K. O. Evans, R. Chandler, and C. Moore for providing reviews of this manuscript. We also thank landowners for providing access to field sites and M. Klinger for data collection. Multiple reviewers and editors helped improve this manuscript.

## Conflict of Interest

None declared.

## Appendix 1

### 1.1

The following R code simulates the latent state of nests, given effects of average.

canopy height (vegetation), generates an encounter history based on observed nests, and then models

daily nest survival according to three measures of nest vegetation: ACH at initiation (Method 1),

ACH upon end of nest attempt (fledged or failed, Method 2), ACH at actual or expected fledging

date (Method 3), and ACH upon end of nest attempt + Initiation date (Method 4). We also included

a null (intercept‐only model), compared models with Akaike's information criterion (AIC), and

evaluated bias in parameter estimates compared with known (simulated) values for effect of ACH.

logit<‐function(x){log(x/(1‐x))} # Function for logit transformation

invlogit<‐function(x){1/(1+exp(‐1*x))} # Function for inverse logit transformation

library(“truncnorm”)

library(“plyr”)

# Logistic exposure link for glm

logexp <‐ function(exposure = 1)

{

linkfun <‐ function(mu) qlogis(mu^(1/exposure))

linkinv <‐ function(eta) plogis(eta)^exposure

mu.eta <‐ function(eta) exposure * plogis(eta)^(exposure‐1) *

.Call(stats:::C_logit_mu_eta, eta, PACKAGE = “stats”)

valideta <‐ function(eta) TRUE

link <‐ paste(“logexp(“, deparse(substitute(exposure)), “)”,

sep=““)

structure(list(linkfun = linkfun, linkinv = linkinv,

mu.eta = mu.eta, valideta = valideta,

name = link),

class = “link‐glm”)

}

seasonLength=60 # Number of days in a season when nests could be initiated

Beta1_ACH<‐0.1 # Effect of average canopy height (nest site vegetation); other values used included

# 0, +/‐ 0.1, +/‐ 0.2, and +/‐ 0.3

survMin<‐0.963 # Mean survival (intercept), for 35% survivorship over 28 days

timeToFledge=28 # Length of nesting period

numNests=600 # Number of nests simulated

skewInitiation=15 # Mean initiation date (0 is middle of season); also simulated skew = 15 for Late nesting

skewEntry=0 # Mean age at entry (detection) of nests (0 is middle of nesting period)

epsilonSD=.05 # Variation in daily nest survival

lastPossibleDate<‐seasonLength+timeToFledge # Length of season under study

expos <‐ 3 # Nest visit interval (days)

# Function to simulate vegetation growth based on the Michaelis–Menten equation

ach.fun<‐function(N){

days<‐seq(1:lastPossibleDate)

ach<‐matrix(NA,nrow=N,ncol=lastPossibleDate)

for (i in 1:N){

a<‐122 + rnorm(1,0,15)

b<‐6 + rnorm(1,0,0.5)

ach[i,]=(a*days)/(b+days)

}

return(ach)

}

# Function to simulate encounter history

encHist<‐function(checkPeriod=expos,nestData){

eh<‐data.frame(ID=NA,expos=NA,vegFail=NA,vegEnd=NA,vegStart=NA,survive=NA,Age=NA,Day=NA,Initiation=NA,trials=1)

numNests<‐dim(nestData)[1]

for (i in 1:numNests){

nestCheck<‐nestData$dayOfEntry[i]+checkPeriod

while (nestCheck < nestData$nestEndDay[i]){

ID<‐nestData$ID[i]

vegStart <‐ nestData$vegStart[i] # Method 1

vegFail <‐ nestData$vegFail[i] # Method 2/4

vegEnd <‐ nestData$vegEnd[i] # Method 3

survive <‐ 1

Day <‐ nestCheck‐expos/2

Age <‐ nestData$ageAtEntry[i] + Day ‐ nestData$dayOfEntry[i]

Initiation <‐ nestData$initiationDates[i] # Method 4

trials <‐ 1

dat<‐cbind(ID,expos,vegFail,vegEnd,vegStart,survive,Age,Day,Initiation,trials)

eh<‐rbind(eh,dat)

nestCheck<‐nestCheck+checkPeriod

}

ID<‐nestData$ID[i]

vegFail <‐ nestData$vegFail[i]

vegEnd <‐ nestData$vegEnd[i]

vegStart <‐ nestData$vegStart[i]

Initiation <‐ nestData$initiationDates[i]

if (nestData$Fail[i]==“Yes”){

survive <‐ 0

} else {survive <‐1}

Day <‐ nestCheck‐expos/2

Age <‐ nestData$ageAtEntry[i] + Day ‐ nestData$dayOfEntry[i]

trials <‐ 1

dat<‐cbind(ID,expos,vegFail,vegEnd,vegStart,survive,Age,Day,Initiation,trials)

eh<‐rbind(eh,dat)

}

return(eh[‐1,])

}

# Simulation code for latent state, then apply encounter history and run candidate models

simulation<‐function(z){

initiationDates<‐as.data.frame(floor(rtruncnorm(numNests,1,seasonLength,mean=(seasonLength/2)+skewInitiation,sd=seasonLength/7)))

colnames(initiationDates)<‐”x”

#ageAtEntry<‐as.data.frame(floor(rtruncnorm(numNests,1,timeToFledge,mean=(timeToFledge/2)+skewEntry,sd=timeToFledge/6)))

ageAtEntry<‐as.data.frame(floor(runif(numNests,1,timeToFledge)))

colnames(ageAtEntry)<‐”x”

dayOfEntry<‐initiationDates+ageAtEntry‐1

fail<‐as.data.frame(rep(x=NA,times=numNests))

nestEndDay<‐as.data.frame(rep(x=0,times=numNests))

possiblyObserved<‐as.data.frame(rep(x=NA,times=numNests))

ach.dat<‐ach.fun(numNests) # Simulate ACH for each nest, over length of the season

ach.mean <‐ mean(ach.dat) # Mean ACH in dataset

ach.sd <‐ sd(ach.dat) # SD of ACH in dataset

ach.dat <‐ (ach.dat‐ach.mean)/ach.sd # Scale ACH for modeling

ID<‐factor(seq(1:numNests))

nestDates<‐cbind(ID,initiationDates,dayOfEntry,ageAtEntry,fail,nestEndDay,possiblyObserved)

colnames(nestDates)<‐c(“ID”,”initiationDates”,”dayOfEntry”,”ageAtEntry”, “Fail”, “nestEndDay”,”possiblyObserved”)

rm(ID,initiationDates,dayOfEntry,ageAtEntry,fail,nestEndDay,possiblyObserved)

# Simulate latent state of each nest

for(k in 1:numNests)

{

for(m in 1:timeToFledge)

{

if((nestDates$dayOfEntry[k])>(nestDates$nestEndDay[k]))

{

nestDates$possiblyObserved[k]<‐”F”

} else{

nestDates$possiblyObserved[k]<‐”T”

}

if(runif(1,0,1) > (invlogit(logit(survMin)+ach.dat[k,nestDates$initiationDates[k]+(m‐1)]*Beta1_ACH)+rnorm(n=1,mean=0,sd=epsilonSD))){

nestDates$Fail[k]<‐”Yes”

nestDates$nestEndDay[k]<‐(nestDates$initiationDates[k]+(m‐1)) # Date of completion of nest attempt

nestDates$vegFail[k]<‐ach.dat[k,nestDates$nestEndDay[k]] # Vegetation on completion date nest failed

nestDates$vegEnd[k]<‐ach.dat[k,nestDates$initiationDates[k]+timeToFledge‐1] # Vegetation on completion date (fledge or fail)

nestDates$vegStart[k]<‐ach.dat[k,nestDates$initiationDates[k]] # Vegetation on initiation date

nestDates$vegFail.us[k]<‐ach.dat[k,nestDates$nestEndDay[k]]*ach.sd+ach.mean # Unscaled estimate of ACH

nestDates$vegEnd.us[k]<‐ach.dat[k,nestDates$initiationDates[k]+timeToFledge‐1]*ach.sd+ach.mean # Unscaled estimate of ACH

nestDates$vegStart.us[k]<‐ach.dat[k,nestDates$initiationDates[k]]*ach.sd+ach.mean # Unscaled estimate of ACH

nestDates$vegFail.diff[k]<‐nestDates$vegFail.us[k]‐nestDates$vegStart.us[k] # Vegetation difference

nestDates$vegEnd.diff[k]<‐nestDates$vegEnd.us[k]‐nestDates$vegStart.us[k] # Vegetation difference

break

} else {

nestDates$Fail[k]<‐”No”

nestDates$nestEndDay[k]<‐(nestDates$initiationDates[k]+(m‐1)) # Date of completion of nest attempt

nestDates$vegEnd[k]<‐nestDates$vegFail[k] # Vegetation on completion date (fledge or fail)

nestDates$vegFail[k]<‐ach.dat[k,nestDates$nestEndDay[k]] # Vegetation on completion date (fledge or fail)

nestDates$vegStart[k]<‐ach.dat[k,nestDates$initiationDates[k]] # Vegetation on initiation date

nestDates$vegFail.us[k]<‐ach.dat[k,nestDates$nestEndDay[k]]*ach.sd+ach.mean # Unscaled estimate of ACH

nestDates$vegEnd.us[k]<‐ach.dat[k,nestDates$initiationDates[k]+timeToFledge‐1]*ach.sd+ach.mean # Unscaled estimate of ACH

nestDates$vegStart.us[k]<‐ach.dat[k,nestDates$initiationDates[k]]*ach.sd+ach.mean # Unscaled estimate of ACH

nestDates$vegFail.diff[k]<‐nestDates$vegFail.us[k]‐nestDates$vegStart.us[k] # Vegetation difference

nestDates$vegEnd.diff[k]<‐nestDates$vegEnd.us[k]‐nestDates$vegStart.us[k] # Vegetation difference

}

}

}

nestDates<‐subset(nestDates, possiblyObserved==“T”) # Retain nests actually observed in study

veg.fail<‐subset(nestDates,Fail==“Yes”)

veg.fledge<‐subset(nestDates,Fail==“No”)

mean.vegend.fail<‐mean(veg.fail$vegEnd.us)

sd.vegend.fail<‐sd(veg.fail$vegEnd.us)

mean.vegend.fledge<‐mean(veg.fledge$vegEnd.us)

sd.vegend.fledge<‐sd(veg.fledge$vegEnd.us)

mean.vegfail.fail<‐mean(veg.fail$vegFail.us)

sd.vegfail.fail<‐sd(veg.fail$vegFail.us)

mean.vegfail.fledge<‐mean(veg.fledge$vegFail.us)

sd.vegfail.fledge<‐sd(veg.fledge$vegFail.us)

mean.vegstart.fail<‐mean(veg.fail$vegStart.us)

mean.vegstart.fledge<‐mean(veg.fledge$vegStart.us)

mean.diff.fail<‐mean(veg.fail$vegFail.diff)

mean.diff.fledge<‐mean(veg.fail$vegEnd.diff)

# Simulate encounter history

nesteh<‐encHist(nestData=nestDates)

# Scale initiation date for Method 4

nesteh$Initiation<‐as.numeric(scale(nesteh$Initiation))

# Formulas for four models: Method 1, Method 2, Method 3, Method 4, and the null

formulas <‐ list(survive/trials ~ vegStart, survive/trials ~ vegFail, survive/trials ~ vegEnd,

survive/trials ~ vegFail + Initiation, survive/trials ~ 1)

aic<‐form <‐ vector(“list”, length = length(formulas))

beta = matrix(NA, nrow=length(formulas),ncol=3)

bias1 = bias2 = numeric(length(formulas))

for (i in 1:4) {

LM <‐ glm(formulas[[i]],data=nesteh,family=binomial(link=logexp(nesteh$expos)))

beta[i,1:length(coef(LM))]<‐ coef(LM)

aic[[i]] <‐ AIC(LM)

bias1[i] <‐ abs(Beta1_ACH‐coef(LM)[2]) # Absolute difference between simulated and estimated effect

# of ACH

bias2[i] <‐ Beta1_ACH‐coef(LM)[2] # Relative bias (positive means underestimated effect, negative

# means overestimated effect)

}

beta[5,1]<‐ coef(glm(formulas[[5]],data=nesteh,family=binomial(link=logexp(nesteh$expos))))

aic[[5]]<‐AIC(glm(formulas[[5]],data=nesteh,family=binomial(link=logexp(nesteh$expos))))

colnames(beta)<‐names(coef(LM))

colnames(beta)[2]<‐”ACH”

# Compare models by Akaike<!‐‐apos‐‐>’<!‐‐/apos‐‐>s Information Criterion (AIC)

test.1v2<‐aic[[1]] < aic[[2]]

test.1v3<‐aic[[1]] < aic[[3]]

test.1v4<‐aic[[1]] < aic[[4]]

test.2v3<‐aic[[2]] < aic[[3]]

test.2v4<‐aic[[2]] < aic[[4]]

test.3v4<‐aic[[3]] < aic[[4]]

test.1vnull<‐aic[[1]] < aic[[5]]

test.2vnull<‐aic[[2]] < aic[[5]]

test.3vnull<‐aic[[3]] < aic[[5]]

test.4vnull<‐aic[[4]] < aic[[5]]

mod.names<‐c(“mod1”,”mod2”,”mod3”,”mod4”,”mod5”)

aic.vls<‐c(aic[[1]],aic[[2]],aic[[3]],aic[[4]],aic[[5]])

aic.df<‐data.frame(mod.names=mod.names,aic.vls=aic.vls)

aic.df$rank<‐rank(aic.df$aic.vls)

out.sim[[z]] <<‐ list(nNests=dim(nestDates)[1],beta=beta,AIC.1v2=test.1v2,AIC.1v3=test.1v3,

AIC.1v4=test.1v4,AIC.2v3=test.2v3,AIC.2v4=test.2v4,AIC.3v4=test.3v4,

aic=aic,bias1=bias1,bias2=bias2,AIC.1vnull=test.1vnull,AIC.2vnull=test.2vnull,

AIC.3vnull=test.3vnull,AIC.4vnull=test.4vnull,rank.mod=aic.df,

mean.vegend.fail=mean.vegend.fail,sd.vegend.fail=sd.vegend.fail,mean.vegend.fledge=mean.vegend.fledge,

sd.vegend.fledge=sd.vegend.fledge,mean.vegfail.fail=mean.vegfail.fail,sd.vegfail.fail=sd.vegfail.fail,

mean.vegfail.fledge=mean.vegfail.fledge,sd.vegfail.fledge=sd.vegfail.fledge,mean.diff.fail=mean.diff.fail,

mean.diff.fledge=mean.diff.fledge,mean.vegstart.fail=mean.vegstart.fail,mean.vegstart.fledge=mean.vegstart.fledge,

initiationDates=mean(nestDates$initiationDates))

}

out.sim<‐ list() # List to store output

n.sim<‐100 # Number of simulated datasets

set.seed(5)

l_ply(seq(1,n.sim),simulation,.progress=“text”)

setwd(“W:/Home/mmcconnell/public/Meghan/PositiveEffect/ACH=0.1/Tests/Round3”)

save(out.sim,file=“R3_Late_28_35.RData”)

# Function to summarize simulation output

sum.true<‐function(x){

n.sim<‐length(x)

test.1v2<‐test.1v3<‐test.1v4<‐test.2v3<‐test.2v4<‐test.3v4<‐test.1vnull<‐test.2vnull<‐test.3vnull<‐test.4vnull<‐numeric(n.sim)

bias1<‐mbias1v1<‐mbias1v2<‐mbias1v3<‐mbias1v4<‐n.Nests<‐numeric(n.sim)

sdbias1v1<‐sdbias1v2<‐sdbias1v3<‐sdbias1v4<‐numeric(n.sim)

bias2<‐mbias2v1<‐mbias2v2<‐mbias2v3<‐mbias2v4<‐numeric(n.sim)

sdbias2v1<‐sdbias2v2<‐sdbias2v3<‐sdbias2v4<‐numeric(n.sim)

rnk_m1<‐rnk_m2<‐rnk_m3<‐rnk_m4<‐rnk_m5<‐numeric(n.sim)

vegstart.fledge<‐vegstart.fail<‐vegend.fail<‐vegend.fledge<‐vegfail.fail<‐vegfail.fledge<‐Initiation<‐numeric(n.sim)

for (i in 1:n.sim){

test.1v2[i]<‐sapply(x[[i]]$AIC.1v2, function(x)sum(grepl(“TRUE”, x)))

test.1v3[i]<‐sapply(x[[i]]$AIC.1v3, function(x)sum(grepl(“TRUE”, x)))

test.1v4[i]<‐sapply(x[[i]]$AIC.1v4, function(x)sum(grepl(“TRUE”, x)))

test.2v3[i]<‐sapply(x[[i]]$AIC.2v3, function(x)sum(grepl(“TRUE”, x)))

test.2v4[i]<‐sapply(x[[i]]$AIC.2v4, function(x)sum(grepl(“TRUE”, x)))

test.3v4[i]<‐sapply(x[[i]]$AIC.3v4, function(x)sum(grepl(“TRUE”, x)))

test.1vnull[i]<‐sapply(x[[i]]$AIC.1vnull, function(x)sum(grepl(“TRUE”, x)))

test.2vnull[i]<‐sapply(x[[i]]$AIC.2vnull, function(x)sum(grepl(“TRUE”, x)))

test.3vnull[i]<‐sapply(x[[i]]$AIC.3vnull, function(x)sum(grepl(“TRUE”, x)))

test.4vnull[i]<‐sapply(x[[i]]$AIC.4vnull, function(x)sum(grepl(“TRUE”, x)))

bias1[i]<‐which(out.sim[[i]]$bias1==max(out.sim[[i]]$bias1))

mbias1v1[i]<‐x[[i]]$bias1[1]

mbias1v2[i]<‐x[[i]]$bias1[2]

mbias1v3[i]<‐x[[i]]$bias1[3]

mbias1v4[i]<‐x[[i]]$bias1[4]

bias2[i]<‐which(out.sim[[i]]$bias2==max(out.sim[[i]]$bias2))

mbias2v1[i]<‐x[[i]]$bias2[1]

mbias2v2[i]<‐x[[i]]$bias2[2]

mbias2v3[i]<‐x[[i]]$bias2[3]

mbias2v4[i]<‐x[[i]]$bias2[4]

rnk_m1[i]<‐ifelse(out.sim[[i]]$rank.mod$rank[1]==1,1,0)

rnk_m2[i]<‐ifelse(out.sim[[i]]$rank.mod$rank[2]==1,1,0)

rnk_m3[i]<‐ifelse(out.sim[[i]]$rank.mod$rank[3]==1,1,0)

rnk_m4[i]<‐ifelse(out.sim[[i]]$rank.mod$rank[4]==1,1,0)

rnk_m5[i]<‐ifelse(out.sim[[i]]$rank.mod$rank[5]==1,1,0)

n.Nests[i]<‐x[[i]]$nNests

vegstart.fledge[i]<‐x[[i]]$mean.vegstart.fledge

vegstart.fail[i]<‐x[[i]]$mean.vegstart.fail

vegend.fledge[i]<‐x[[i]]$mean.vegend.fledge

vegend.fail[i]<‐x[[i]]$mean.vegend.fail

vegfail.fledge[i]<‐x[[i]]$mean.vegfail.fledge

vegfail.fail[i]<‐x[[i]]$mean.vegfail.fail

Initiation[i]<‐x[[i]]$initiationDates

}

sum.t.1v2<‐sum(test.1v2[])/n.sim

sum.t.1v3<‐sum(test.1v3[])/n.sim

sum.t.1v4<‐sum(test.1v4[])/n.sim

sum.t.2v3<‐sum(test.2v3[])/n.sim

sum.t.2v4<‐sum(test.2v4[])/n.sim

sum.t.3v4<‐sum(test.3v4[])/n.sim

sum.t.1vnull<‐sum(test.1vnull[])/n.sim

sum.t.2vnull<‐sum(test.2vnull[])/n.sim

sum.t.3vnull<‐sum(test.3vnull[])/n.sim

sum.t.4vnull<‐sum(test.4vnull[])/n.sim

bias1v1<‐length(which(bias1[]==1))/n.sim

bias1v2<‐length(which(bias1[]==2))/n.sim

bias1v3<‐length(which(bias1[]==3))/n.sim

bias1v4<‐length(which(bias1[]==4))/n.sim

meanbias1v1<‐mean(mbias1v1[])

meanbias1v2<‐mean(mbias1v2[])

meanbias1v3<‐mean(mbias1v3[])

meanbias1v4<‐mean(mbias1v4[])

sdbias1v1<‐sd(mbias1v1[])

sdbias1v2<‐sd(mbias1v2[])

sdbias1v3<‐sd(mbias1v3[])

sdbias1v4<‐sd(mbias1v4[])

RMSE1v1<‐sqrt(sum(mbias1v1^2))

RMSE1v2<‐sqrt(sum(mbias1v2^2))

RMSE1v3<‐sqrt(sum(mbias1v3^2))

RMSE1v4<‐sqrt(sum(mbias1v4^2))

bias2v1<‐length(which(bias2[]==1))/n.sim

bias2v2<‐length(which(bias2[]==2))/n.sim

bias2v3<‐length(which(bias2[]==3))/n.sim

bias2v4<‐length(which(bias2[]==4))/n.sim

meanbias2v1<‐mean(mbias2v1[])

meanbias2v2<‐mean(mbias2v2[])

meanbias2v3<‐mean(mbias2v3[])

meanbias2v4<‐mean(mbias2v4[])

sdbias2v1<‐sd(mbias2v1[])

sdbias2v2<‐sd(mbias2v2[])

sdbias2v3<‐sd(mbias2v3[])

sdbias2v4<‐sd(mbias2v4[])

RMSE2v1<‐sqrt(sum(mbias2v1^2))

RMSE2v2<‐sqrt(sum(mbias2v2^2))

RMSE2v3<‐sqrt(sum(mbias2v3^2))

RMSE2v4<‐sqrt(sum(mbias2v4^2))

avg_rnk_m1<‐mean(rnk_m1[])

avg_rnk_m2<‐mean(rnk_m2[])

avg_rnk_m3<‐mean(rnk_m3[])

avg_rnk_m4<‐mean(rnk_m4[])

avg_rnk_m5<‐mean(rnk_m5[])

mean.vegstart.fledge<‐mean(vegstart.fledge)

mean.vegstart.fail<‐mean(vegstart.fail)

mean.vegend.fledge<‐mean(vegend.fledge)

mean.vegend.fail<‐mean(vegend.fail)

mean.vegfail.fledge<‐mean(vegfail.fledge)

mean.vegfail.fail<‐mean(vegfail.fail)

sd.vegstart.fledge<‐sd(vegstart.fledge)

sd.vegstart.fail<‐sd(vegstart.fail)

sd.vegend.fail<‐sd(vegend.fledge)

sd.vegend.fledge<‐sd(vegend.fail)

sd.vegfail.fail<‐sd(vegfail.fledge)

sd.vegfail.fledge<‐sd(vegfail.fail)

return(data.frame(test1v2=sum.t.1v2,test1v3=sum.t.1v3,test1v4=sum.t.1v4,test2v3=sum.t.2v3,test2v4=sum.t.2v4,test3v4=sum.t.3v4,

test1vnull=sum.t.1vnull,test2vnull=sum.t.2vnull,test3vnull=sum.t.3vnull,test4vnull=sum.t.4vnull,

maxbias1v1=bias1v1,maxbias1v2=bias1v2,maxbias1v3=bias1v3,maxbias1v4=bias1v4,meanbias1v1=meanbias1v1,

meanbias1v2=meanbias1v2,meanbias1v3=meanbias1v3,meanbias1v4=meanbias1v4,sdbias1v1=sdbias1v1,sdbias1v2=sdbias1v2,

sdbias1v3=sdbias1v3,sdbias1v4=sdbias1v4,maxbias2v1=bias2v1,maxbias2v2=bias2v2,maxbias2v3=bias2v3,maxbias2v4=bias2v4,

meanbias2v1=meanbias2v1,meanbias2v2=meanbias2v2,meanbias2v3=meanbias2v3,meanbias2v4=meanbias2v4,sdbias2v1=sdbias2v1,

sdbias2v2=sdbias2v2,sdbias2v3=sdbias2v3,sdbias2v4=sdbias2v4,RMSE1v1=RMSE1v1,RMSE1v2=RMSE1v2,RMSE1v3=RMSE1v3,RMSE1v4=RMSE1v4,

RMSE2v1=RMSE2v1,RMSE2v2=RMSE2v2,RMSE2v3=RMSE2v3,RMSE2v4=RMSE2v4,

avg_rnk_m1=avg_rnk_m1,avg_rnk_m2=avg_rnk_m2,avg_rnk_m3=avg_rnk_m3,avg_rnk_m4=avg_rnk_m4,

avg_rnk_m5=avg_rnk_m5,max.n=max(n.Nests),

min.n=min(n.Nests),mean.n=mean(n.Nests),mean.vegstart.fledge=mean.vegstart.fledge,

mean.vegstart.fail=mean.vegstart.fail,mean.vegend.fail=mean.vegend.fail,mean.vegend.fledge=mean.vegend.fledge,

mean.vegfail.fledge=mean.vegfail.fledge,mean.vegfail.fail=mean.vegfail.fail,sd.vegstart.fledge=sd.vegstart.fledge,

sd.vegstart.fail=sd.vegstart.fail,sd.vegend.fail=sd.vegend.fail,sd.vegend.fledge=sd.vegend.fledge,

sd.vegfail.fledge=sd.vegfail.fledge,sd.vegfail.fail=sd.vegfail.fail,mean.Initiation=mean(Initiation),

sd.Initiation=sd(Initiation)))

}

sum.true(out.sim)

end<‐sum.true(out.sim)
